# Proteomic Signatures of the Zebrafish (*Danio rerio*) Embryo: Sensitivity and Specificity in Toxicity Assessment of Chemicals

**DOI:** 10.1155/2010/630134

**Published:** 2010-10-14

**Authors:** Karen Hanisch, Eberhard Küster, Rolf Altenburger, Ulrike Gündel

**Affiliations:** Department of Bioanalytical Ecotoxicology, Helmholtz Centre for Environmental Research-UFZ, Permoser Straße 15, 04318 Leipzig, Germany

## Abstract

Studies using embryos of the zebrafish *Danio rerio* (*Dar*T) instead of adult fish for characterising the (eco-) toxic potential of chemicals have been proposed as animal replacing methods. Effect analysis at the molecular level might enhance sensitivity, specificity, and predictive value of the embryonal studies. The present paper aimed to test the potential of toxicoproteomics with zebrafish eleutheroembryos for sensitive and specific toxicity assessment. 2-DE-based toxicoproteomics was performed applying low-dose (EC_10_) exposure for 48 h with three-model substances Rotenone, 4,6-dinitro-o-cresol (DNOC) and Diclofenac. By multivariate “pattern-only” PCA and univariate statistical analyses, alterations in the embryonal proteome were detectable in nonetheless visibly intact organisms and treatment with the three substances was distinguishable at the molecular level. Toxicoproteomics enabled the enhancement of sensitivity and specificity of the embryonal toxicity assay and bear the potency to identify protein markers serving as general stress markers and early diagnosis of toxic stress.

## 1. Introduction

 As fish are highly developed vertebrates in the aquatic ecosystems, they are of major relevance in ecotoxicology mainly as important model organisms for toxicity assessment of water pollution. The acute fish test, for example, [1] is among the base test set necessary for regulatory risk assessment of chemicals. Among fish, the zebrafish (*Danio rerio*), a small tropical fish native to rivers of India and South Asia [2], has emerged as a popular vertebrate model in (eco-) toxicology [3–5] because it is unique with respect to the level of available knowledge and technology and has many benefits like its rapid development, easy maintenance in the laboratory, large number of offspring, and access to experimental manipulation [6]. As the use of fish embryos is considered a refinement, if not replacement of animal experiments [7, 3R principle] and due to the available knowledge of developmental processes of zebrafish, Nagel [5] has introduced the embryo test with the zebrafish *Danio rerio* test on teratogenicity (*Dar*T) as an alternative to the acute fish toxicity test. The *Dar*T analyses acute toxicity in embryos by screening lethal effects, developmental disorders, and other morphological, sublethal endpoints. Next to animal replacement, studies on embryonal stages offer some advantages compared to adults. Test organisms can be obtained in high numbers and at short breeding time, cultivation is less cost and time consuming, organisms are of small size and require no feeding. In Germany, the *Dar*T has already replaced the acute fish tests for toxicity assessment of waste water effluents [8] but is not yet extended for use in chemical risk assessment [9]. Improvement of knowledge about toxic responses regarding sensitivity, specificity, and novel biomarkers increasing the predictive value for possible long-term effects in the embryonic model system [10] would help to advance DarT also for chemical testing. 

One possibility to address these challenges might be the analysis of effects at the molecular level [11]. Genomic studies on early life stages of the zebrafish and the highly advanced sequencing of the zebrafish genome (http://www.sanger.ac.uk/Projects/D_rerio/) has lead to the proposal extending the *Dar*T assay to the Mol*Dar*T or “Gene-*Dar*T” (gene expression *Danio rerio* embryo test) [12–15]. This approach is based on studying gene expression profiles as additional toxicological endpoints and combines both early effect diagnosis and mode of action analysis [16].

The present paper aims to extend the *Dar*T assay in terms of a proteome *Danio rerio* embryo assay (“Pro*Dar*T”) analysing effects at the proteome and thus functional level in zebrafish embryos. In environmental monitoring, proteomics has been successfully applied to show sensitive and specific toxicity-related responses in the protein profiles of mussels [17]. Until today, only few proteomics approaches on developing zebrafish with toxicological background [18–20] have been published. One of the major drawbacks is the high abundance of yolk proteins (Vitellogenins, Vtgs) early in development of oviparous animals, which can mask and reduce the sensitivity for the detection of changes in the cellular protein pattern. Ziv et al. [21] have studied the proteome of zebrafish oocytes, at a time point at which few yolk proteins have been inoculated in the eggs. Other researchers have applied techniques to manually remove the yolk sacs in early embryos [22, 23]. However, these techniques may interfere with the detection of stress-induced responses by the tested compounds and Vtgs could still be identified after manual deyolking [23]. In the present paper, eleutheroembryos (i.e., hatched but not yet free feeding embryos) are applied since a strong decrease in yolk and yolk proteins has been reported [19, 23–25]. As eleuthero-embryos have all organs developed, are not surrounded by a chorion, show increased activities of detoxification processes [26] and are free swimming, toxicodynamic, and kinetic, toxicity processes in eleuthero-embryos might be more similar to adults compared to embryos. To contribute to the further advance of using fish embryos as a method for replacement of experiments with adult fish, a two-dimensional-gel electrophoresis-(2-DE) based proteomics approach with 5 days old zebrafish eleuthero-embryos was applied in the present study to (i) investigate toxicity-related responses in the embryonic protein profiles, (ii) characterise the potential for sensitive and specific effect assessment, and (iii) look for candidate protein biomarkers reflecting the organism health status at the molecular level.

For this purpose, effects to the eleutheroembryonal proteome caused by low effect concentrations of three model substances, which have been detected in environment and have different mode of action, two pesticides Rotenone and 4,6dinitro-o-cresol (DNOC) and the drug Diclofenac, were studied.

## 2. Experimental Procedures

### 2.1. Fish Culture, Embryo Collection, and Eleutheroembryo Bioassay

WIK (Wild-type India Kalcutta) zebrafish were obtained from the Tübingen Zebra Fish Stock Centre at the Max Planck Institute for Developmental Biology and cultivated as described in Küster [27]. The eleuthero-embryo bioassays were carried out based on the OECD Draft Guideline for testing of chemicals (OECD 2006) with small modifications due to physicochemical characteristics of model substances (see below). 

79 hpf old eleuthero-embryos were incubated for 48 h in the exposure solutions at static conditions (temperature 27 ± 1°C, 12 h light/dark with 30 mmol photons m^2^ s^1^). 

The model substances were Rotenone (CAS RN 83-79-4), 4,6-dinitro-o-cresol (DNOC, CAS RN 534-52-1), and Diclofenac-sodium salt (CAS RN 15307-79-6). All substances were of analytical grade and purchased from Riedel de Haen (Rotenone and DNOC) and MP Biomedicals (Diclofenac), respectively. 

To estimate concentration-response relationships, the model substance Rotenone was tested in the concentration range of 0.25 nM to 2.5 *μ*M, DNOC from 0.05 to 50 *μ*M, and Diclofenac between 1.4 and 200 *μ*M (nominal concentrations). Constant concentrations are assumed during the 48 h exposure time with applied conditions for Diclofenac as exposure concentrations were demonstrated to be very stable at the above test conditions (U. Krug, pers. communication). Tests with DNOC were done according to OECD guideline, that is, by using adhesive foils and an additional lid to cover the multiwell plates to decrease possible evaporation. The assay for Rotenone was carried out in glass vials (instead of multiwell plates made from polystyrene) to avoid concentration decrease during exposure due to expected sorption because of the log K_OW_ of 4.1 of Rotenone (EpiSuite Vers. 4.0) as recommended by Riedl et al. [28] and Schreiber et al. [29].

Due to the effect concentrations used, it was not possible to check for the stability of the low exposure concentrations with the currently available analytical methods. Because of that, no statements can be made about the real concentrations. As stated by [28] Riedl and Altenburger (2007) “In ISO 14442 (2004), volatile substances are characterized by a Henry's constant of H ≥ 1 Pa m^3^ mol^−1^ (log K_AW_ = −3.4), highly volatile substances by H ≥ 100 Pa m^3^ mol^−1^ (log K_AW_ = −1.4). Loss of exposure concentration due to lipophilicity is expected for hydrophobic compounds with an octanol/water partition coefficient of log KOW > 4 (OECD SERIES ON TESTING AND ASSESSMENT Number 23, 2000).” Only Rotenone and DNOC were seen as critical in terms of their physicochemical characteristics. So, possible losses in the test system were tried to counteract by the use of glass (lipophilic Rotenone, log K_OW_ 4.1) and covering (low volatility of DNOC, Henry constant of 1.4 × 10^−6^ atm m^3^/mol) to decrease the loss of substances to the maximum. 

The stock solutions for the proteomic experiment were the same as the ones used for the concentration-response relationships. So, although the real concentrations are not known, the concentrations related to the effect level are similar. After exposure, all eleuthero-embryos were analysed for lethal and sublethal effects by inverse microscopy (50x magnification, Olympus IX70-S8F, Hamburg, Germany). Coagulation, absent blood circulation and absent heartbeat are considered as lethal toxicological endpoints and were used to estimate concentration-response relationships using a logistic model (*y* = 100 + (−100)/(1 + (*x*/*x*0)  p), with *x* being the concentration in % (v/v), *x*0 the median effect (EC_50_) and *p* as the slope) (ORIGIN software, version 6.0, Friedrichsdorf, Germany). 

The estimated EC_10_ concentrations of these concentration-response relationships were used as the exposure concentration in the following proteome analysis (EC_10,  Rotenone_ = 0.05 *μ*M; EC_10,  DNOC_ = 2.80 *μ*M; EC_10,  Diclofenac_ = 12.60 *μ*M). For the proteomics experiments, 79 hpf old eleuthero-embryos were collectively exposed for 48 h in 200 mL glass beakers with a density of one organism per 2 mL exposure solution. In the control experiments, 45 individuals were incubated in 90 mL of ISOwater (ISO 7346-3). The exposure to the model substances was done with 50 individuals in 100 mL each. After exposure, the eleuthero-embryos, which did show visible effects were discarded and the left over intact organisms (at least 40 per replicate) were pooled in 2 mL reaction tubes and the samples were washed four times with 1 mL of *aqua dest.* Finally, the embryos were shock frozen in liquid nitrogen and stored at −80°C until analysis.

### 2.2. Two-Dimensional Gel Electrophoresis

The 2-DE sample preparation was done according to Gündel et al. [19], where proteins were extracted using 0.25 mL lysis buffer (8 M Urea, 2% CHAPS, 0.5 % v/v IPGBuffer 47 linear (GE Healthcare, Uppsala, Sweden) and 1% protease inhibitor cocktail (SIGMA, Taufkirchen, Germany) for each sample (40 pooled embryos). The subsequent 2DE was carried out according to Görg et al. [30]. The isoelectric focusing (IEF) was performed using the Ettan IPGphor (GE Healthcare) and 18 cm linear, pH 47 immobiline dry strips (GE Healthcare). Rehydration was carried out at room temperature overnight with 400 *μ*L rehydration solution *per* dry strip (DeStreak solution (GE Healthcare), 0.5% v/v IPGBuffer 4–7 linear (GE Healthcare)). Fifty micrograms of protein were subjected to IEF *via* cup loading on the cathode and the focussing was performed under following conditions: 150 V, 2 h; 300 V, 2 h; 600 V, 2 h; 8000 V gradient, 0.5 h; 8000 V, 11 h; all steps at 20°C. After IEF, the immobiline dry strips were equilibrated at room temperature according to Gündel et al. [19]. For the polyacrylamide gel electrophoresis (SDS-Page), as the second dimension, the IPG strips were sealed on the top of 1 mm thick 14% polyacrylamide gels (Ettan DALT 12, GE Healthcare). Vertical electrophoresis was carried out overnight (18 h) at 12°C and about 1 W/gel. The gels were silver stained according to Heukeshoven and Dernick [31] and Yan et al. [32] and digitalized at a resolution of 200 dpi using the image scanner (GE Healthcare, Uppsala, Sweden). Subsequently, they were air dried at room temperature between two cellophane sheets (BioRad, Munich, Germany).

### 2.3. Image Analysis and Statistics

Densitometric image analysis for the 2DE gels was performed with the software package Delta 2D (Version 3.4, DECODON GmbH, Germany). The 100% matching strategy was chosen for spot detection. A “fusion” gel of the experiment was created after gel matching of all replicate 2D-gels and all proteins were detected and edited on this virtual “fusion” gel. Subsequently, the detected spot pattern of this fusion gel was transferred to all other gels in the experiment. This method ensures the same spot detection pattern on each gel in one experiment. The amount of protein present in a single spot was described as the spot volume, that is, the area of the spot multiplied by the pixel density. Individual spot volumes were normalised to the total protein amount (e.g., all protein spots added up together) detected within each gel and the amount of each spot was therefore expressed as a relative volume. All proteome analyses were run in triplicates. Due to the inherent semiquantitative silver staining method, the 10% largest protein spots were excluded from the normalisation set. 

Multivariate pattern and univariate spot-to-spot-methods were applied for statistical analysis. Principal component analysis (PCA), including all detected proteins in one experiment, was performed with the software package Jmp (Version 7.0, Cary, USA) to compare protein patterns on the gels. Univariate spot-to-spot analysis was performed using a Student's *t*-test. Hereby, only spots were considered as up- or downregulated proteins, which showed an at least twofold difference in abundance compared to controls and which were significantly different from controls with a *P* < .05 or <.01 (highly significant).

### 2.4. Trypsin Digestion and Identification of Proteins

Proteins of interest were excised from the stained gels. Following silver removal, the spots were subjected to in-gel trypsin digestion as previously described in Benndorf et al. [33] Peptides were reconstituted in 0.1% formic acid, injected by an autosampler and were concentrated on a trapping column (nanoAcquity UPLC column, C18, 180 *μ*m × 2 cm, 5 *μ*m, Waters, Eschborn, Germany) with water containing 0.1% formic acid at flow rates of 15 *μ*L/min. After 4 min, the peptides were eluted onto the separation column (nanoAcquity UPLC column, C18, 75 *μ*m × 250 mm, 1.7 *μ*m, Waters, Eschborn, Germany). Chromatography was performed by using 0.1% formic acid in solvents A (100% water) and B (100% acetonitrile), with peptides eluted over 30 min with a 8%–40% solvent B gradient using a nano-HPLC system (nanoAcquity, Waters) coupled to an LTQ-Orbitrap mass spectrometer (Thermo Fisher Scientific). Continuous scanning of eluted peptide ions was carried out between 150–2000 m/z, automatically switching to MS/MS CID mode on ions exceeding an intensity of 2000. Raw MS/MS spectra were converted to mgf-files using the ProteomDiscoverer 1.0 (Thermo Fisher Scientific). MS data were submitted to the online Mascot (http://www.matrixscience.com, may 2009) and searched against *Danio rerio* (Taxonomy ID: 7955) in the National Centre for Biotechnology Information nucleotide database (NCBInr, may 2009; 70,034 protein entries) tolerating up to two tryptic missed cleavages, a mass tolerance of 10 ppm for precursor ions, 0.5 Da for MS/MS product ions allowing for methionine oxidation (dynamic modification) and cysteine carbamido methylation (static modification). For the Mascot threshold, a probability score of 5% was applied (significance threshold: *P* ≤ .05). Hereby, the cutoff score value for accepting individual MS/MS spectra was set to 0 to ensure high-sequence coverage. The identified proteins were characterised *via* the Swiss-Prot and TrEMBL (http://www.expasy.org/sprot) databases. All molecular weights (MWs) were calculated by the online PROTPARAM tool (http://www.expasy.org/tools/protparam.html).

## 3. Results

### 3.1. Optimisation of 2-DE Method Concerning Eleutheroembryo Age

The high abundance and high number of yolk vitellogenin (Vtgs) derivatives in protein samples of developing oviparous organisms, like the zebrafish, is a major drawback when lower abundant proteins are to be studied. The known reduction of yolk-to-cell mass during development [19, 23] was the basis to address this drawback in the present study. Five days postfertilisation (120 hpf), zebrafish have developed to eleuthero-embryo stages with a strongly reduced yolk, developed mouth and are able to start external feeding. Several authors have shown that at that time of development the zebrafish proteome is not dominated by Vtgs anymore [19, 23, 25]. However, at 120 hpf, still some Vtg fragments could be identified in the protein samples as is shown in Supplementary Material available at doi:10.1155/2010/630134 (SM, Table 1) and it can be concluded that yolk utilisation is still not at an end at the fifth day of development. But seven days after fertilisation eleuthero-embryos starve when not fed.

**Figure 1:**
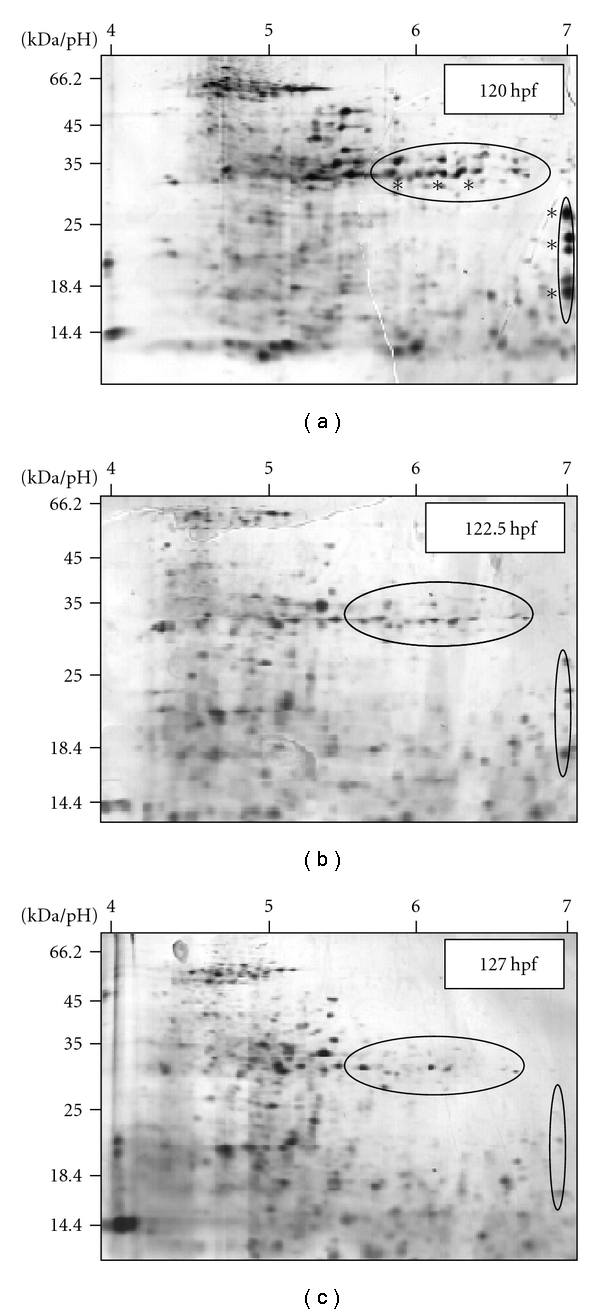
Changes in proteome patterns of eleuthero-embryos at three different time points (a) at 120 hpf, (b) at 122.5 hpf and (c) at 127 hpf. Areas with most obvious changes are encircled. Number and abundance of proteins in these areas clearly decrease over time. Proteins identified as vitellogenin derivatives are labelled by asterisks (∗).

Preceding the toxicoproteomics studies, the optimal sampling time point of eleuthero-embryos combining both, a low number of yolk proteins in the protein samples and the avoidance of starvation stress, had to be found. To characterise the abundance of yolk proteins in the larval proteome, samples deriving from eleuthero-embryos at three different time points (120 hpf, 122.5 hpf and 127 hpf) were studied (Figure 1). To avoid starvation, no samples from eleuthero-embryos older than 127 hpf were characterised. Although most proteins in the three protein samples show constant abundance at the different measured time points, at least two areas on the gels presented in Figures 1(a)–1(c) were quite variable. A strong decrease of the number and amount of the detected proteins in these areas was observed. In previous studies, a couple of proteins of these areas were identified as Vtg derivatives [19]. As the number and abundance of these Vtg proteins have strongly reduced in samples from 127 hpf old eleuthero-embryos (Figure 1(c)), eleuthero-embryos were sampled at this age/time point for all the following proteomic analyses.

### 3.2. Effects of DNOC, Rotenone and Diclofenac on Treated Eleutheroembryos at Phenotypic Level

The choice of relevant concentrations for the toxicoproteomics experiments with the three tested chemicals was based on the previous characterisation of effects at higher levels of biological organisation (morphological, physiological effects) in the eleuthero-embryos. Effect detection in eleuthero-embryos was done similar to the *Dar*T [5] in terms of test regime, exposure time and toxic endpoints. The 48-hour-long exposure in the toxicant solutions started at around 79 hpf when most of the eleuthero-embryos did hatch.

**Figure 2:**
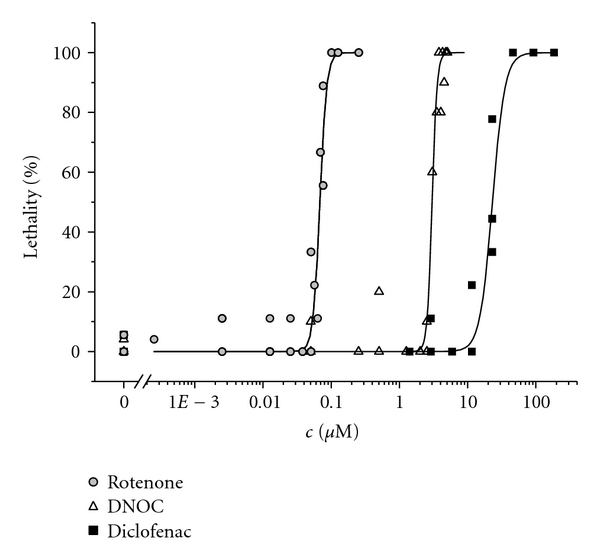
Microscopically visible lethal effects of Rotenone, DNOC and Diclofenac to eleuthero-embryos studied after 48 h exposure. Concentration-effect relationships based on a logistic model are shown. Parameter estimates were as follows: Rotenone: EC_50_  [*μ*M] = 0.068 ± 0.00, *p* = 8.750 ± 2.25, DNOC: EC_50_ [*μ*M] = 3.007 ± 0.05, *p* = 12.311 ± 1.80, Diclofenac: EC_50_ [*μ*M] = 23.076 ± 1.08, *p* = 4.746 ± 1.65.

All three substances caused concentration-dependent lethal effects in the organisms. Concentration-effect relationships based on lethal endpoints are shown in Figure 2. The potency of Rotenone (EC_50  _= 0.068 ± 0.00 *μ*M) was strongest compared to DNOC (EC_50  _= 3.0 ± 0.05 *μ*M) and Diclofenac (EC_50  _= 23 ± 1.08 *μ*M). The slopes obtained for the modelled concentration-effect relationships for Rotenone (8.75 ± 2.25) and DNOC (12 ± 1.80) were quite steep and exceeded the one estimated for Diclofenac (4.75 ± 1.65) exposure in eleuthero-embryos. Rotenone and DNOC solely caused coagulation of the organisms, whereas diverse effects in the eleuthero-embryos were detected after exposure to Diclofenac. These effects included coagulation and lethal impairments in the cardiovascular system but also, to a minor extent, oedema in heart and yolk region or malformations of the backbone. 

### 3.3. Toxicoproteomics with Zebrafish Eleutheroembryos Using the Three Toxicants Rotenone, DNOC, and Diclofenac

Rotenone, DNOC and Diclofenac were selected as model substances with different modes of action to study the potential of proteomics for sensitive and specific effect detection. Although they have different molecular toxicity targets, both, Rotenone and DNOC, affect the respiratory chain and were chosen because of their action in an important primary metabolic pathway. The insecticide Rotenone binds, as a primary toxicity target, to the PSST-subunit of complex 1 of the electron chain and inhibits the oxidative phosphorylation [34]. The insecticide and herbicide DNOC acts as a decoupler of the mitochondrial oxidative phosphorylation by decoupling of the electron transport process from ATP synthesis [35]. The pharmaceutical Diclofenac, as an anti-inflammatory drug, inhibits the cyclooxygenases COX1 and COX2 [36] and was chosen as a toxicant affecting a secondary biochemical pathway by inhibiting the prostaglandin synthesis.

For all three substances, the modelled low-dose EC_10_ concentrations were selected for the proteome studies (EC_10  Rotenone_ = 0.05 *μ*M, EC_10  DNOC_ = 2.80 *μ*M, EC_10  Diclofenac_ = 12.60 *μ*M). To avoid interpretation problems due to aggregating effects at the protein level for affected and nonaffected embryos, only those exposed eleuthero-embryos were included in the 2DE experiments that did not show physiological or morphological effects. This procedure should allow the detection of stress induced changes at the molecular level prior the occurrence of microscopically visible effects. 

**Figure 3:**
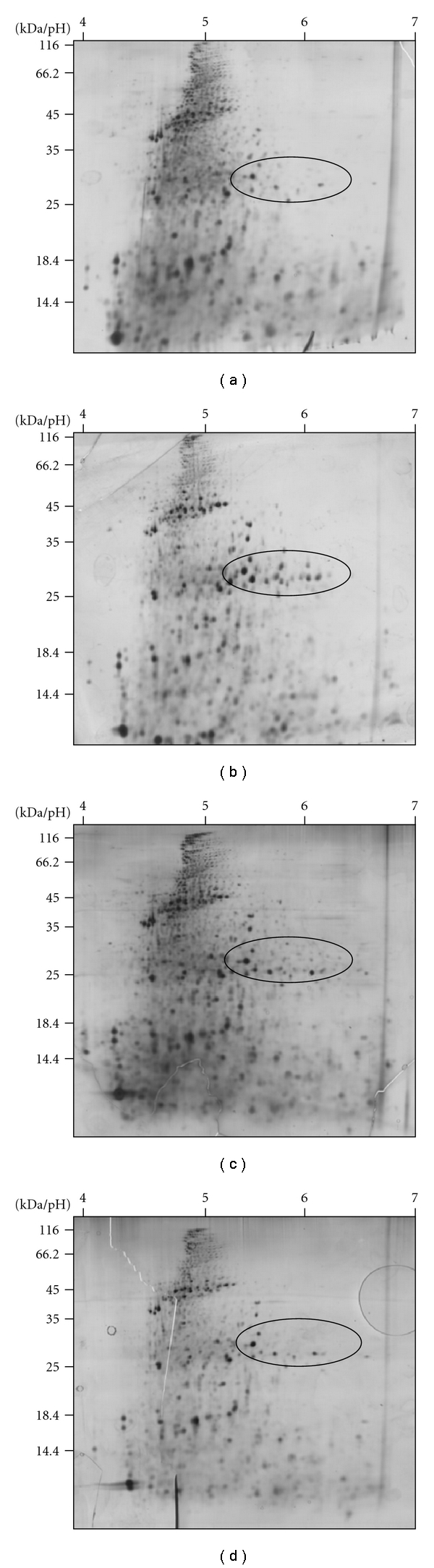
2-DE gels from proteomics experiments: (a) control conditions, (b) EC_10_ treatment with Rotenone, (c), EC_10_ treatment with DNOC and (d) EC_10_ treatment with Diclofenac. All protein samples were separated under the same 2-DE conditions: Immobiline strips pH 4–7 in the first and 14% PAA gels in the second dimension.

In Figure 3, the typical proteome pattern from control eleuthero-embryos (a) and eleuthero-embryos treated with the EC_10_ concentrations of Rotenone (b), DNOC (c) and Diclofenac (d) are depicted. Clear differences between the samples and controls are indicated by encircled areas. Two statistical approaches, based on univariate and multivariate analyses tools were applied for the detection of toxicity related changes in the eleuthero-embryo proteome [37]. 

#### 3.3.1. Principal Component Analysis (PCA)

**Figure 4:**
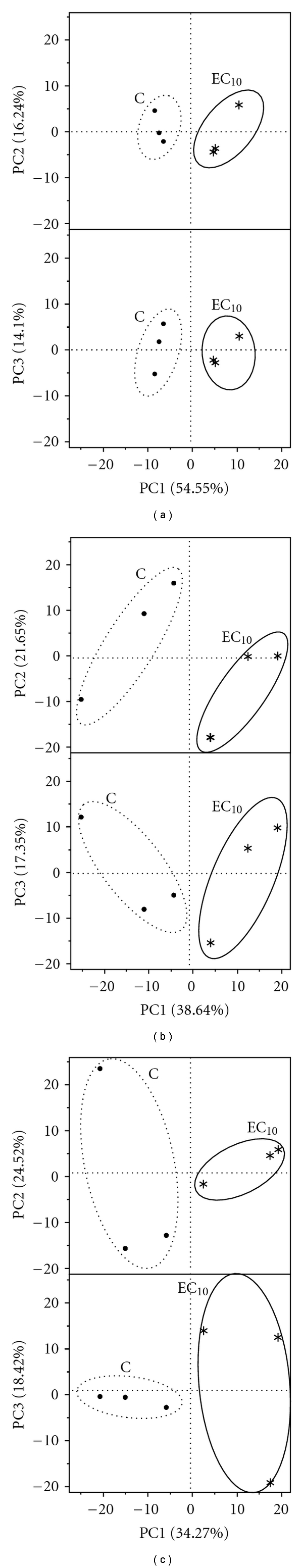
Principal component analysis scores (PC1, PC2, and PC3) for protein pattern assessment of control and treated eleutheroembryos with (a) Rotenone, (b) DNOC, and (c) Diclofenac. Control groups are indicated with c. Three independent replicates were performed for all treatment groups (EC_10_ treatment with either Rotenone, DNOC, or Diclofenac).

It was performed to analyse the whole proteome pattern of exposed and nonexposed organisms. The received PCA scores for the tested toxicants are shown in Figure 4. The PCA distinguished between control and treatment groups for all three substances. 

For all chemicals, the first component (PC1) provided separation between the control and treatment groups (Figures 4(a)–4(c)). PC2 and PC3, in contrast, did not contribute information to distinguish between control- and exposure- protein patterns but accounted for variances within the replicates. The percentage contributions for PC1, PC2, and PC3 for each toxicant are shown in Figure 4.

#### 3.3.2. Univariate Spot-to-Spot Analysis

**Figure 5:**
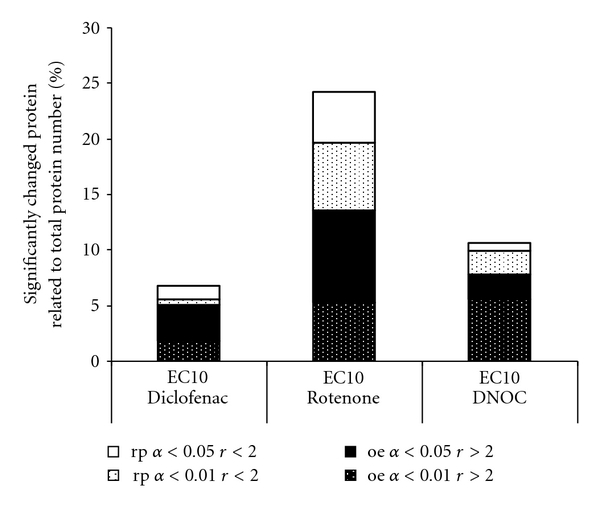
Summary of results from spot-to-spot analysis of proteomics experiments with Diclofenac (Dic), Rotenone(R), and DNOC (D). For all tested concentrations the percentages of protein spots, related to all detected protein spots in the 2-DE experiment, are depicted that show significant (*P* < .05) or highly significant changes (*P* < .01) and a minimum of twofold changed expression levels compared to controls. Proteins showing an at least twofold increase in abundance compared to control (*r* < 2) are considered as upregulated (oe), all proteins showing an at least twofold decrease in abundance compared to controls are considered as downregulated (rp).

It was performed as a way of analysing the 2-DE experiments to obtain detailed information about single proteins changed in expression or abundance after treatment. Results from univariate analysis are shown in Figure 5 and Table 1.

Overall, Rotenone (EC_10_) caused the highest percentage of significantly changed proteins (24.1%), followed by DNOC (EC_10_) with 10.8% and by Diclofenac (EC_10_) with 6.8%. This order is not changed if only the high-significant changed proteins (*P* < .01) are considered (indicated in Figure 5 and Table 1). The results from univariate spot-to-spot analysis mirror the results from the multivariate analysis showing a sharp separation between control and treatment protein pattern for Rotenone, less clear separation of the treatment conditions for DNOC and low difference between control and treatment groups for Diclofenac (Figure 4). The spot IDs, relative volumes, standard distributions and Student's *t*-test results for all differentially expressed proteins obtained from treatment with the single substances are shown on 2-DE gels and tables in the Supplemental Material (Figures SM1 and SM2, Tables 3–5).

### 3.4. Comparison of Results from Toxicoproteomics Experiments with Rotenone, DNOC, and Diclofenac

**Figure 6:**
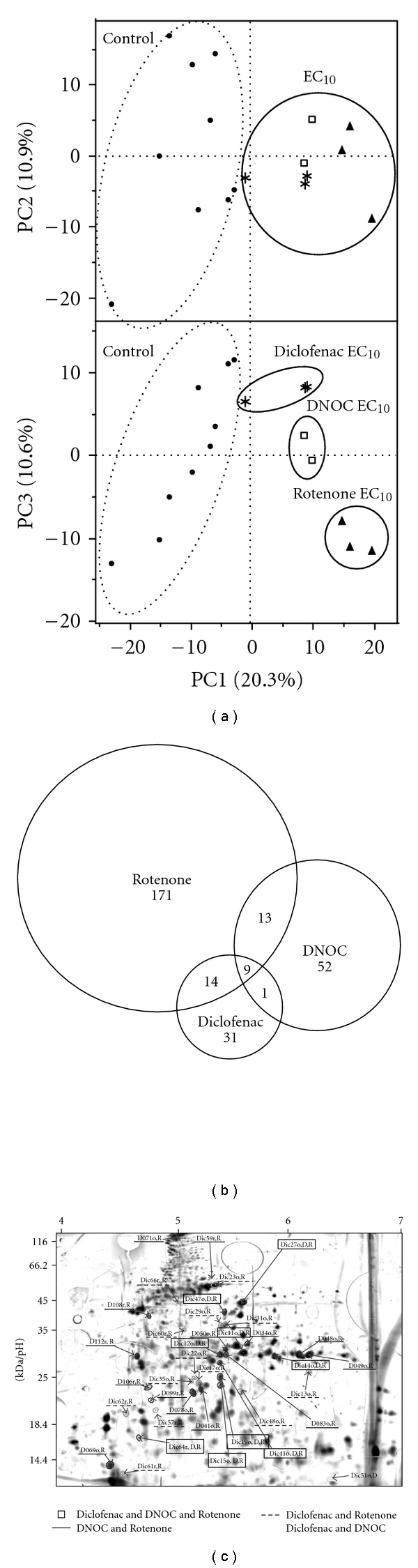
Comparison of results from toxicoproteomics experiments with Rotenone, DNOC, and Diclofenac exposure. (a) PCA scores when all samples are included in PCA analysis. (b) Venn-Diagramm based on results from substance-specific univariate spot-to-spot analysis. Most of the protein spots changed specifically. 9 proteins concurrently changed after Rotenone, DNOC, and Diclofenac treatment. Moreover, 13 proteins simultaneously changed after Rotenone and DNOC treatment, and 14 proteins showed changed expression levels at exposure against Diclofenac or Rotenone. Only one protein was found to be changed in the larval protein samples after DNOC as well as Diclofenac treatment. (c) 2-DE gel from eleutheroembryonal protein samples. All proteins that significantly changed in expression in at least two toxicoproteomics experiments (treatment with either Rotenone, DNOC, or Diclofenac) are labelled: Protein labels of protein spots simultaneously altered in Rotenone, DNOC, and Diclofenac experiments are framed (□), in Rotenone and DNOC experiments are underlined (—), in Rotenone and Diclofenac experiments are dashed (—), and in DNOC, and Diclofenac experiments are labelled by name only.

Changes in the eleuthero-embryo proteome pattern from all treatment conditions (Rotenone, DNOC and Diclofenac) were compared to enable the differentiation between unspecific from model substance specific reactions. Comparison was realised on pattern-(Figure 6(a)) and individual-protein level (Figures 6(b) and 6(c)). 

All obtained control protein patterns could be clearly distinguished from treatment situations by principal component 1 (Figure 6(a)) whereas PC2 separated controls belonging to different experiments. PC1 also provided a separation between proteome patterns of Rotenone treated eleuthero-embryos and the other treatments. PC3 sorted the protein patterns according to all three different treatment groups (Figure 6(a)). These results were confirmed considering the comparison at the individual protein level, which is demonstrated in the Venn diagram in Figure 6(b). So, each model substance caused its own set of differentially expressed proteins. 

However, next to substance specific changed proteins also proteins were detected, which showed changed expression levels independent of substance identity. These are indicated on the 2DE gel in Figure 6(c) and in the Supplemental Material (SM, Table 2). Nine proteins could be detected, which simultaneously changed expression levels after Rotenone, DNOC, and Diclofenac treatment. These might be a base set for the development of general stress biomarkers for the indication of exposure. This was confirmed by first identification results of those proteins that had been associated with stress by Monsinjon and Knigge [37] (Table 2).

## 4. Discussion

Improvement of knowledge about toxic responses and regarding information on sensitivity and specificity of effect assessment might contribute to the advance of the zebrafish embryo test for testing of chemicals as animal replacement method for regulatory purposes. The aim of the present paper was to characterise the potential of proteomics with zebrafish eleuthero-embryos for sensitive and specific effect assessment of chemical exposure, which, to our knowledge, has not been studied so far. Hereby, the main questions to be answered were whether toxicity related responses can be detected at low-dose ranges in the proteome profiles of treated eleuthero-embryos, whether exposure with different model substances can be discriminated at the proteome level and whether possible candidate protein biomarkers for predictive effect diagnosis might be proposed. Therefore, proteomics was established for zebrafish eleuthero-embryos and proteomic analyses were performed with three different compounds, including two insecticides, affecting primary metabolic pathways (oxidative phosphorylation), Rotenone and DNOC [38, 39], and the pharmaceutical Diclofenac, with an anti-inflammatory mode of action [36].

The results will be discussed in two parts. Firstly, methodological aspects including eleuthero-embryo age and the applied model substances are discussed. In the second part, the discussion is related to the results of the proteomic experiments.

### 4.1. Methodological Aspects

#### 4.1.1. Eleutheroembryo Age

In contrast to adult animals, proteome analysis of developing organisms addresses a biological system that is highly variable in terms of physiological, morphological, and other parameters over time, which is very likely to be mirrored at the molecular level. Hence, the exact sampling point of eleuthero-embryos for the proteomics studies is of concern. Eleuthero-embryos were sampled at 127 hpf for the proteome analyses for two reasons. On the one hand, the observed shift of the 2DE protein patterns from yolk proteins towards cellular proteins during embryonal development [19, 23] is nearly completed at this developmental stage and Vtg rich areas on the gels are not predominant anymore. On the other hand, 127 hpf old eleuthero-embryos are vital and do not show signs of starvation, which could be assumed if yolk is nearly utilised and no external feeding of the organisms would take place. To our knowledge, the point of time of complete yolk consumption in *Danio rerio* has not been investigated so far. However, yolk utilisation may strongly depend on many parameters including movements of the eleuthero-embryos, temperature or light conditions. These parameters might vary slightly for each biological sample deriving from eleuthero-embryos and could lead to differences in the proteome pattern. Hence, for all performed proteomic experiments controls from the same spawning event as the treated samples were included. This is also proposed for any future applications of proteomics with zebrafish eleuthero-embryos.

#### 4.1.2. Concentrations Tested

The output of a proteomics experiment strongly depends on the applied model substance concentrations and exposure times and should be interpreted in relation to observed effects at higher biological organisation levels in the analysed organisms. As a shortcoming we have to state that it was not possible to quantify the exact concentrations mainly due to the low concentrations of two of the substances (EC_10_), the volumes used and the respective limits of quantification. In spite of the above, all effects on higher organisation levels like morphological, physiological or behavioural effects are preceded by effects at the molecular level [11]. Proteomics investigations at substance concentrations or exposure times that do not cause any microscopically visible effects in the organisms may lead to the identification of proteins involved in the primary response or adaptation processes after exposure [17, 18, 40]. This could be used for prediction of effects at higher organisation levels [37]. However, without relation to effects at the phenotypic level, the selection of concentrations or exposure times causing detectable relevant effects at the proteome level might be difficult. But the discussion of exposure concentrations in relation to concentration dependent phenotypic effects for the proteomics experimental setup has not been focus of many ecotoxicoproteomics studies so far [17]. The investigation of low-effect concentrations like EC_10_ as done here with a proteomics approach in intact organisms enables both, the testing of a toxicity relevant concentration and the analyses of molecular effects, which precede phenotypic physiological or morphological effects.

### 4.2. Proteomics Experiments: Sensitivity and Specificity of Responses

#### 4.2.1. Sensitivity

One question of the present study was whether toxic stress can be detected in the proteome of treated zebrafish eleuthero-embryos that do not show a microscopically visible damage. By application of multivariate and univariate methods for the analysis of the proteomics data, proteome pattern of control and treated but intact eleuthero-embryos could clearly be distinguished at low sublethal concentrations of Rotenone, DNOC and Diclofenac. It can be concluded that the proteomics approach for all analysed substances lead to detection of a response at the molecular level in eleuthero-embryos not showing microscopically visible lesions. This would confirm the suggested scope of toxicogenomics approaches [11, 37, 40] to sensitively detect effects at the molecular level prior to the occurrence of effects at the phenotypic level and the definition of marker proteins, which might be used for predictive effect diagnosis [41].

#### 4.2.2. Specificity

The characterisation of the specificity of the detected response to treatments with different substances with the applied proteomics approach was a further major concern in the present study. In terms of specificity, the established *Dar*T assay sometimes has limitations [6, 10]. The results of the *Dar*T as performed in this study confirm such concerns as less specific reactions were detectable at the phenotypic level after Rotenone, DNOC and Diclofenac treatment. Observed effects caused by the three different substances were quite similar and coagulation was predominant. Early responses at the molecular level are supposed to be more related to substance specific effects [11] that could also give hints to mode of action and mechanisms of toxicity of the tested substances [41]. This is supported by PCA based pattern analyses clearly distinguishing between proteomes of eleuthero-embryos treated with the different model substances. Moreover, each tested substance caused its own specific pattern of changed proteins and hence, its own protein expression signature (PES). The term PES was introduced by Bradley and coworkers [42, 43] to define a set of proteins differing between contaminant exposure and control. Shrader et al. [20] demonstrated the applicability for PES to distinguish between different exposure scenarios with endocrine-disruption in zebrafish embryos. By studying mussels from different polluted field sites, Knigge et al. [44] also described a subset of proteins forming a classifier to distinguish between polluted and unpolluted situations. PES can enable the identification of substance specific biomarker patterns, which are considered to provide an overcome of the uncertainties associated with the extraction of single protein markers as described by Knigge et al. [44] and Monsinjon and Knigge [37]. This was also shown in a toxicogenomic study that described specific gene expression profile pattern in zebrafish embryos enabling the discrimination of exposure against 11 model compounds [16] or by a toxicoproteomics study of marine pollutants on mussels [17].Hence, with our results having identified specific protein patterns for each exposure scenario would support the concept of PES and the idea of extracting information for certain exposure scenarios solely from changed protein patterns without the need to identify single proteins.

The PES of each substance was mainly determined by the number of changed proteins. For all three substances the same effect concentration, which lead to microscopically visible effects in 10% of the treated organisms (EC_10_), was tested with the toxicoproteomic approach. At the molecular level, however, in terms of the number of differentially expressed proteins, differences in the effect levels could be detected for all three substances. At EC_10_ concentration, Rotenone caused the change of about 24% of all detected proteins, DNOC of about 11% and Diclofenac of about 7% of all proteins. So, the observed effect level at the molecular level differs from the effect level derived from analysis of visible toxic endpoints at the phenotypic level but, interestingly, correlated well with the determined potencies of the substances. Most detected proteins from whole embryonic fish proteomic studies are likely to belong to high-abundant protein classes such as cellular organisation or metabolic pathways. These have been previously associated with toxic stress [37]. Thus, it might be stated that the number of changed proteins in ecotoxicoproteomics studies of whole organisms might correlate with the stress or decompensation status of the organism.

Analysing the number of changed proteins the effect analysis at the proteome level could give information on how basal the affected metabolic pathways are in contrast to classical toxic endpoints. Rotenone and DNOC have a mode of action in a primary metabolic pathway, the oxidative phosphorylation [38, 39]. Both substances caused an effect on a higher number of proteins in the proteome compared to Diclofenac with the primary mode of action in a secondary biochemical pathway (inhibition of cyclooxygenases) [36]. Direct impairments in energy metabolism affect many cellular processes and enzymes and are in direct relation to changed rates of biosyntheses, as general protein biosynthesis, all of which uses up ATP. Hence, changes of many proteins in the larval proteome after Rotenone or DNOC treatment may well be considered as plausible. For Rotenone, this was also confirmed by a proteomics study from Jin et al. [45] who identified 110 significantly changed mitochondrial proteins in Rotenone exposed dopameric cell lines. The reversibility of mode of action from Rotenone and DNOC and the multiple other actions described for Rotenone [46–48] might be consulted for explaining the higher number of changed proteins after Rotenone treatment compared to DNOC. Future protein identifications would help to obtain closer insights in mechanisms of toxicity of the tested substances but were not within the scope of this study.

#### 4.2.3. General Stress Markers

Although specific PES were detectable for Rotenone, DNOC and Diclofenac, spot-to-spot analyses revealed 9 proteins that showed concurrently and significantly changed expression levels after treatment with all substances. Apraiz and co-workers [17] have introduced the term “minimal PES” for the set of generally responding proteins. These proteins seem to respond to a wider variety of toxic stress and might provide an origin for the development of unspecific biomarkers distinguishing between control and exposure scenarios. 

Identification results have shown that one of these proteins matched to vitellogenin sequences. Vitellogenins are yolk proteins that serve in embryonic nutrition and which decrease in abundance during development [19, 23]. An increased abundance of those vitellogenins (Vtg) in exposed eleuthero-embryos might indicate retardation in development of the exposed eleuthero-embryos at the molecular level, which can hardly be followed with microscopy based methods. As the detected Vtg protein is a Vtg fragment, endocrine disrupting processes, which have been also associated with (full-length) Vtg expression [15] are not assumed here. 

In addition, three cytoskeleton proteins (myosin, actin_,_ and tubulin) were identified. This is in accordance to other toxicoproteomics studies, which have found cytoskeleton proteins associated with toxic responses [17, 37]. The cytoskeleton has been proposed to be one of the first targets of oxidative stress [49] and Apraiz et al. [17] have extensively discussed the expression change of tubulin, which they found to be part of the minimal PES after exposure of mussels to three different chemicals. Shi et al. [18] also described that cytoskeleton maintenance was predominantly affected in zebrafish larvae after PFOS exposure and Manduzio et al. [50] found altered expression of actins and myosins in mussels as indication for water pollution. Although, there is also criticism about housekeeping proteins to be good marker proteins [37], our results support studies, which have shown that toxic exposure might lead to significant changes in abundance of cytoskeleton proteins. These could serve as markers to monitor the health status of an organism.

*β*-crystallin also responded to exposure with all three substances. Crystallins are the dominant structural components of the vertebrate eye lens and alteration in its expression might be connected to disturbed embryonal eye development. A connection of toxic exposure and lens degeneration in fish has been recently published [51] Moreover, Shi et al. [18] have detected a correlation of *γ*-crystalline expression and toxic stress in protein profiles of PFOS treated zebrafish larvae.

It can be concluded that the established toxicoproteomics approach with zebrafish eleuthero-embryos enabled the detection of candidate protein markers indicating developmental impairments and toxic stress at the molecular level prior the manifestation of visible lesions. Perceivable next steps will concentrate on assay development such as enzyme assays or Western blots for some of the found protein markers. This enables the analysis of robustness and exposure-time and exposure-concentration dependence of the found protein signals after exposure to chemical stress and to characterise their potential to be stress biomarkers in* Danio rerio *embryos.

## 5. Conclusions

Proteomics was established for eleuthero-embryos of the zebrafish (*Danio rerio*). With univariate and multivariate statistical analysis tools, the potential of this approach was confirmed to sensitively detect effects in organisms treated with low toxicant concentrations of the model substances Rotenone, DNOC, and Diclofenac. The different exposure scenarios could be distinguished at the molecular level as each substance caused its own protein expression signature. Moreover, it was shown that proteomic investigations hold the possibility to detect candidate protein markers that might serve as general stress markers indicating the health status of an organism and be usable for early diagnosis of toxic stress. Thus, an extension of *Dar*T to the study of effects at the proteome level (Pro*Dar*T) promises to allow a more sensitive, specific and refined toxicity assessment.

## Supplementary Material

Table 1: Identification of protein spots in the protein pattern of 5 days old zebrafish larvae.
Table 2: Protein spots changed in expression after exposure to EC10 of rotenone, DNOC and diclofenac in two or all three exposure experiments.
Table 3: Regulated protein spots after exposure of 3 days old zebrafish eleutheroembryos for 48 h to the EC10 of rotenone. 207 spots changed in expression, 116 of these protein spots were up-regulated and 91 down-regulated.
Table 4: Regulated protein spots after exposure of 3 days old zebrafish eleutheroembryos for 48 h to the EC10 of DNOC. 76 spots changed in expression, 56 of these protein spots were up-regulated and 20 down-regulated.
Table 5: Regulated spots after exposure of 3 days old zebrafish eleutheroembryos for 48 h to the EC10 of diclofenac. 47 spots changed in expression, 42 of these protein spots were up-regulated and 5 down-regulated.
Figure SM1: Comparison of the proteome pattern of eleutheroembryos of the zebrafish exposed to rotenone (A) DNOC (B) and diclofenac (C) with the respective EC10 concentration (rotenone = 0.05 *μ*M; DNOC = 2.80 *μ*M; Diclofenac = 12,6 *μ*M). Only in expression upregulated proteins are depicted. The x-axis shows the pH-range and the y-axis the molecular weight (MW) in kDa. Exposed to rotenone, 116 proteins, to DNOC, 56 proteins and to Diclofenac, 41 proteins are increased in their intensity. Modified proteins are encircled and labelled with numbers. The labelled spots can be found in the supplementary material section.
Figure SM2: Comparison of the proteome pattern of eleutheroembryos of the zebrafish exposed to rotenone (A), DNOC (B) and diclofenac (C) with the respective EC10 concentration (rotenone = 0.05 *μ*M; DNOC = 2.80 *μ*M; diclofenac = 12,6 *μ*M). Only repressed proteins are depicted. The x-axis shows the pH-range and the y-axis the molecular weight (MW) in kDa. Exposed to rotenone, 91 proteins, to DNOC, 20 proteins and to diclofenac, 14 proteins are decreased in their intensity. Modified proteins are encircled and labelled with numbers. The labelled spots can be found in supplementary material section.

## Figures and Tables

**Table 1:** Summary of results from spot-to-spot analysis for all performed toxicoproteomics experiments: Diclofenac (EC_10_), Rotenone (EC_10_) and DNOC (EC_10_). Numbers and percentage (in relation to total protein number on the gel) of significantly (*P* < .05) and highly-significantly (*P* < .01) at least twofold changed proteins are indicated in the table. Data are sorted for up-regulated (oe, ratio sample/control >2) and down-regulated (rp, ratio sample/control <2) proteins. In addition, the sum or percentage of all changed proteins for each condition is shown (rp and oe).

Toxi Compound	Total number of Proteins detected on the gels	*P* value	rp [number of proteins]	rp [%]	oe [number of proteins]	oe [%]	rp and oe [number of proteins]	rp and oe [%]
Diclofenac	814	0.05	14	1.7	41	5.0	55	6.8
	814	0.01	4	0.5	15	1.8	19	2.3
Rotenone	860	0.05	91	10.6	116	13.5	207	24.1
	860	0.01	39	4.5	68	8.4	107	12.9
DNOC	703	0.05	20	2.8	56	8.0	76	10.8
	703	0.01	5	0.7	17	2.4	22	3.1

**Table 2:** Summary of positive identification of protein spots changed in expression after exposure to EC_10_ concentrations of Rotenone (R), DNOC (D) and Diclofenac (Dic) to zebrafish eleuthero-embryos.

Spot	Mascot Score	Protein Name	UniProtKB Accession	Peptides assigned	Sequence coverage [%]	MW_obs/cal_ [kDa]	pI_obs/cal_	Ratio Rotenone	Ratio DNOC	Ratio Diclofenac
Dic11o, D, R	430.29	vitellogenin 1	Q1LWN2	12	8.7	30.0/128.0	5.6/8.68	13.6	4.1	2.4
Dic14o, D, R	94.0	LOC553473 (*β*-crystallin)	Q502C7	5	10.8	24.5/27.6	5.9/7.67	47.3	3.1	3.0
Dic27o, D, R	197.79	actin, alpha, cardiac muscle 1a	Q6IQR3	10	23.1	45.0/42.0	5.6/5.22	3.3	3.6	2.3
Dic47o, D, R	644.34	tubulin, beta 2c	Q6P5M9	25	25.8	44.0/49.8	5.5/4.79	40.6	12	26.5
Dic64r, D, R	321.38	myosin, light polypeptide 2	O93409	14	50.3	17.0/18.9	4.7/4.39	0.4	0.5	0.5

## Abbreviations

COX: Cyclooxygenase
*Dar*T: Zebra fish embryo toxicity test
2DE: Two-dimensional gel electrophoresis
DNOC: Dinitro-*o*-cresol
Dpf: Days post fertilisation
EC: Effect concentration
Hpf: Hours post fertilisation
ISO: International Standard Organisation
Lv: Lipovitellin
MW: Molecular weight
oe: Up-regulated
PCA: Principal component analysis
PES: Protein expression signature
Pro*Dar*T: Toxicoproteomics with embryos of *Danio rerio *
Pv: Phosvitin
rp: downregulated
SM: Supplementary material
Vtg: Vitellogenin
