# Supplementary material for: Proteomic Signatures of the Zebrafish (Danio rerio) Embryo: Sensitivity and Specificity in Toxicity Assessment of Chemicals
Source: Int J Proteomics. 2010 Oct 14;2010:630134. doi: 10.1155/2010/630134 (PMC3200224; doi:10.1155/2010/630134)
Supplement: Supplementary file 1 — Table 1: Identification of protein spots in the protein pattern of 5 days old zebrafish larvae. Table 2: Protein spots changed in expression after exposure to EC10 of rotenone, DNOC and diclofenac in two or all three exposure experiments. Table 3: Regulated protein spots after exposure of 3 days old zebrafish eleutheroembryos for 48 h to the EC10 of rotenone. 207 spots changed in expression, 116 of these protein spots were up-regulated and 91 down-regulated. Table 4: Regulated protein spots after exposure of 3 days old zebrafish eleutheroembryos for 48 h to the EC10 of DNOC. 76 spots changed in expression, 56 of these protein spots were up-regulated and 20 down-regulated. Table 5: Regulated spots after exposure of 3 days old zebrafish eleutheroembryos for 48 h to the EC10 of diclofenac. 47 spots changed in expression, 42 of these protein spots were up-regulated and 5 down-regulated. Figure SM1: Comparison of the proteome pattern of eleutheroembryos of the zebrafish exposed to rotenone (A) DNOC (B) and diclofenac (C) with the respective EC10 concentration (rotenone = 0.05 μM; DNOC = 2.80 μM; Diclofenac = 12,6 μM). Only in expression upregulated proteins are depicted. The x-axis shows the pH-range and the y-axis the molecular weight (MW) in kDa. Exposed to rotenone, 116 proteins, to DNOC, 56 proteins and to Diclofenac, 41 proteins are increased in their intensity. Modified proteins are encircled and labelled with numbers. The labelled spots can be found in the supplementary material section. Figure SM2: Comparison of the proteome pattern of eleutheroembryos of the zebrafish exposed to rotenone (A), DNOC (B) and diclofenac (C) with the respective EC10 concentration (rotenone = 0.05 μM; DNOC = 2.80 μM; diclofenac = 12,6 μM). Only repressed proteins are depicted. The x-axis shows the pH-range and the y-axis the molecular weight (MW) in kDa. Exposed to rotenone, 91 proteins, to DNOC, 20 proteins and to diclofenac, 14 proteins are decreased in their intensity. Modified pro [file 630134.f1.pdf]

Supplementary Material

CONTENT

Table 1 (Picture??) Identification of protein spots in the protein pattern of 5 days old zebrafish larvae.

Table 2 Protein spots changed in expression after exposure to EC_{10} of rotenone, DNOC and diclofenac in two or all three exposure experiments.

Table 3 Regulated protein spots after exposure of 3 days old zebrafish eleutheroembryos for 48 h to the EC_{10} of rotenone. 207 spots changed in expression, 116 of these protein spots were up-regulated and 91 down-regulated.

Table 4 Regulated protein spots after exposure of 3 days old zebrafish eleutheroembryos for 48 h to the EC_{10} of DNOC. 76 spots changed in expression, 56 of these protein spots were up-regulated and 20 down-regulated.

Table 5 Regulated spots after exposure of 3 days old zebrafish eleutheroembryos for 48 h to the EC_{10} of diclofenac. 47 spots changed in expression, 42 of these protein spots were up-regulated and 5 down-regulated.

Figure SM1 see PP file

Figure SM2 see PP file

TABLES

Table 1

Identification of protein spots in the protein pattern of 5 days old zebrafish eleutheroembryos.

MASCOT-score	*Protein-NAME*	*$MW_{obs/cal}$ [kDa]*	*$pI_{obs/cal}$*
172	vitellogenin 2	48.0 / 69.0	6.4 / 7.84
752.33	vitellogenin 1	37.0/128.0	5.5/8.68
68	vitellogenin 1	47.0 / 14.9	6.6 / 8.74
431.1	vitellogenin 1	33.0/128.0	5.7/8.68
>50	vitellogenin 1	30.0/128.0	6.1/8.68
>50	vitellogenin 1	30.0/128.0	6.2/8.68
106	tubulin alpha	41.0 / 50.0	5.5 / 4.93
139	tubulin	33.0 / 52.5	5.0 / 4.9
380	troponin c	17.0 / 18.2	4.0 / 3.96
253	tropomyosin	36.0 / 28.6	4.6 / 4.74
128	tropomyosin	40.0 / 32.7	4.5 / 4.7
237	ribosomal protein large PO (RpIpO)	33.0/34.4	4.8/5.73
196	Proteasome subunit alpha type 5	26.0/26.4	4.7/4.74
93	myosin	16.5 / 16.5	4.6 / 4.39
394	myosin	15.0 / 16.5	4.4 / 4.39
346	myosin	30.0 / 22.0	4.7 / 5.54
293	myosin	12.0 / 18.8	4.5 / 4.68

174	myosin	14.0 / 16.8	4.4 / 4.39
147	myosin	32.0 / 48.6	4.9 / 5.52
120	myosin	30.0 / 48.6	5.1 / 5.52
631	krt4	43.0 / 53.9	5.1 / 5.34
513	Keratin 5	44.5 / 59.0	5.1 / 5.34
711	Keratin 4	44.0 / 54.0	5.4 / 5.34
121	Keratin 5	44.0 / 58.6	4.9 / 5.34
85	hyp. Protein	19.0/82.9	5.9/5.43
101	hyp protein LOC641320	30.0/41.8	5.4/5.3
432/401	hyp protein LOC323055/ Apolipoprotein	42.6/31.7	5.1/4.95
359	hyp protein LOC447930	19.0 / 19.1	4.6 / 4.64
170	hsp60	55.0 / 61.1	5.4 / 5.56
210	cytoceratin	44.0 / 46.7	4.7 / 5.13
613	ATP-Synthase	48.0 / 54.9	5.2 / 5.35
353	ATP-Synthase	55.0 / 54.9	5.2 / 5.25
462	Apolipoprotein A-IV	24.5 / 30.0	4.6 / 4.82
66	alpha cardiac actin	43.0/ 41.9	5.9 / 5.29
236	alpha cardiac actin	37.0 / 42.0	5.5 / 5.29
132	alpha actin 1	44.0 / 41.9	5.1 / 5.23
374	alpha actin	40.0 / 42.0	5.5 / 5.18
1110.35	actin. alpha 1. skeletal muscle	55.0/42.0	5.4/5.23
280	actin. alpha 1	30.0 / 41.9	5.5 / 5.23
99	actin alpha	31.0 / 42.0	5.4 / 5.23
673	actin	45.0 / 41.7	5.3 / 5.3
291	actin	40.0 / 41.9	5.6 / 5.23

Table 2

Protein spots changed in expression after exposure to EC_{10} of rotenone, DNOC and diclofenac in two (DicR/DR/DicD) or all three exposure experiments (DicDR).

Rotenon	DNOC	Diclofenac	Name in Figures
R037o	D042o	Dic27o	DicDR1
R049o	D079o	Dic15o	DicDR2
R068o	D051o	Dic41o	DicDR3
R071o	D043o	Dic12o	DicDR4
R088o	D044o	Dic11o	DicDR5
R107o	D055o	Dic19o	DicDR6
R109o	D035o	Dic47o	DicDR7
R111o	D040o	Dic14o	DicDR8
R189r	D101r	Dic64r	DicDR9
R021o		Dic13o	DicR1
R060o		Dic23o	DicR2
R069o		Dic48o	DicR3
R072o		Dic17o	DicR4
R085o		Dic29o	DicR5
R087o		Dic31o	DicR6
R132r		Dic35o	DicR7
R134r		Dic59r	DicR8
R139r		Dic61r	DicR9
R140r		Dic60r	DicR10
R150r		Dic62r	DicR11
R168r		Dic66r	DicR12

R173r		Dic22o	DicR13
R186r		Dic57r	DicR14
R013o	D078o		DR1
R014o	D050o		DR2
R036o	D071o		DR3
R041o	D083o		DR4
R044o	D041o		DR5
R081o	D049o		DR6
R096o	D034o		DR7
R145r	D106r		DR8
R161r	D069o		DR9
R169r	D108r		DR10
R175r	D099r.R		DR11
R196r	D112r		DR12
R75o	D048o		DR13
	D062o	Dic51o	DicD1

Table 3

Regulated protein spots after exposure of 3 days old zebrafish eleutheroembryos for a duration of 48 h to the EC_{10} concentration of rotenone. 207 spots changed in expression. 116 of these protein spots were up-regulated and 91 down-regulated.

Spot	Mean C	Rsd [%]	Mean EC_{10}	Rsd [%]	ratio	t-Test	Spot	Mean C	Rsd [%]	Mean EC_{10}	Rsd [%]	ratio	t-Test
R001o	0.19	35.1	0.38	11.6	2.0	97.4	R036o	0.13	32.3	0.42	15.4	3.3	99.4
R002o	0.01	24.5	0.03	16.8	2.1	97.6	R037o	0.27	45.4	0.91	8.7	3.3	99.6
R003o	0.65	21.7	1.37	19.5	2.1	97.1	R038o	0.02	65.4	0.08	11.1	3.5	99.0
R004o	0.57	46.8	1.21	6.9	2.1	96.8	R039o	0.13	29.5	0.44	23.7	3.5	98.4
R005o	0.08	24.6	0.16	9.5	2.1	99.2	R040o	0.05	21.6	0.18	30.3	3.5	97.0
R006o	0.17	15.2	0.36	9.4	2.1	99.7	R041o	0.09	44.3	0.38	18.7	4.1	99.2
R007o	0.20	12.4	0.43	5.5	2.2	99.9	R042o	0.02	49.3	0.06	10.8	4.1	99.7
R008o	0.42	9.5	0.93	6.3	2.2	99.9	R043o	0.00	67.9	0.02	32.1	4.2	96.0
R009o	0.18	11.9	0.40	20.2	2.2	98.0	R044o	0.12	39.2	0.52	11.4	4.3	99.8
R010o	0.07	35.3	0.15	20.6	2.2	96.2	R045o	0.07	39.6	0.31	5.6	4.3	99.9
R011o	0.27	20.4	0.63	12.2	2.3	99.4	R046o	0.01	70.8	0.06	9.1	4.5	99.6
R012o	0.20	10.9	0.45	12.7	2.3	99.6	R047o	0.04	11.5	0.17	19.7	4.6	99.5
R013o	0.19	29.3	0.45	25.1	2.3	95.5	R048o	0.03	59.4	0.14	13.1	4.7	99.6
R014o	0.15	12.7	0.36	11.9	2.3	99.6	R049o	0.06	39.6	0.29	19.3	4.8	99.4
R015o	0.10	58.2	0.23	8.7	2.3	96.3	R050o	0.02	50.7	0.09	4.0	4.8	99.9
R016o	0.16	19.4	0.38	13.9	2.4	99.3	R051o	0.03	30.1	0.16	36.4	4.9	96.2
R017o	0.03	40.0	0.07	4.1	2.4	99.1	R052o	0.03	102.5	0.16	23.7	5.0	97.8
R018o	0.04	2.6	0.10	16.8	2.4	99.2	R053o	0.03	58.1	0.19	33.6	5.7	97.1
R019o	0.53	7.2	1.30	19.1	2.5	98.8	R054o	0.04	21.5	0.22	24.7	5.7	99.0
R020o	0.03	14.8	0.08	15.1	2.5	99.4	R055o	0.09	55.9	0.51	11.3	5.7	99.9
R021o	0.06	58.4	0.15	6.2	2.5	97.6	R056o	0.03	56.7	0.16	26.3	5.9	98.6
R022o	0.15	21.9	0.39	29.9	2.6	95.1	R057o	0.12	70.4	0.72	3.9	5.9	99.9
R023o	0.26	30.5	0.67	9.8	2.6	99.5	R058o	0.00	141.4	0.01	23.0	5.9	97.6

R024o	0.08	59.8	0.21	4.6	2.7	98.2	R059o	0.02	30.6	0.11	39.7	6.0	95.8
R025o	0.02	11.5	0.05	13.0	2.7	99.7	R060o	0.12	29.6	0.73	21.8	6.1	99.4
R026o	0.01	35.8	0.03	12.0	2.8	99.3	R061o	0.02	52.6	0.12	34.0	6.4	97.3
R027o	0.08	37.9	0.23	13.4	2.8	99.1	R062o	0.03	92.1	0.16	22.7	6.4	98.9
R028o	0.03	27.3	0.10	20.5	2.8	98.4	R063o	0.05	34.3	0.32	9.2	6.4	100.0
R029o	0.01	34.8	0.03	30.4	2.9	95.2	R064o	0.04	72.9	0.24	18.6	6.5	99.5
R030o	0.30	70.1	0.87	13.4	2.9	97.1	R065o	0.01	87.6	0.04	40.3	6.7	95.3
R031o	0.03	17.8	0.09	8.0	2.9	99.9	R066o	0.00	87.2	0.02	24.0	6.7	98.8
R032o	0.05	69.3	0.16	19.5	3.0	96.5	R067o	0.02	57.0	0.11	35.1	6.8	97.1
R033o	0.05	34.6	0.14	26.7	3.0	96.9	R068o	0.06	67.6	0.44	9.1	7.3	99.9
R034o	0.08	49.4	0.24	23.2	3.1	97.2	R069o	0.08	58.8	0.58	14.2	7.5	99.8
R035o	0.11	48.6	0.35	1.5	3.2	99.7	R070o	0.01	28.0	0.10	15.3	7.9	99.9
R071o	0.11	38.4	0.98	9.4	8.6	100.0	R113o	0.01	85.0	0.95	10.5	134.9	100.0
R072o	0.07	14.4	0.67	19.7	9.0	99.7	R114o	0.00	141.4	0.11	17.0	652.5	99.9
R073o	0.01	72.2	0.10	6.8	9.1	100.0	R115o	0.00	141.4	0.07	49.1	950.0	95.5
R074o	0.01	81.9	0.11	37.8	9.1	96.8	R116o	0.02	24.2	0.00	141.4	0.0	99.6
R075o	0.08	34.7	0.73	9.3	9.2	100.0	R117r	0.04	18.2	0.00	141.4	0.0	99.8
R076o	0.03	78.9	0.26	39.6	9.3	96.4	R118r	0.55	43.1	0.01	141.4	0.0	96.8
R077o	0.02	114.6	0.21	5.9	9.6	99.9	R119r	0.08	41.6	0.00	141.4	0.0	97.2
R077o	0.02	114.6	0.21	5.9	9.6	99.9	R120r	0.10	22.1	0.00	93.2	0.0	99.7
R078o	0.05	41.9	0.52	24.2	9.7	99.3	R121r	0.01	35.8	0.00	141.4	0.0	98.2
R079o	0.01	82.0	0.11	41.6	10.3	96.1	R122r	0.06	18.3	0.00	97.7	0.1	99.8
R080o	0.01	95.0	0.07	7.0	10.7	100.0	R123r	0.29	15.1	0.02	31.6	0.1	99.9
R081o	0.08	57.9	0.91	2.9	10.9	100.0	R124r	0.08	23.6	0.01	78.5	0.1	99.4
R082o	0.00	59.2	0.01	35.2	11.5	97.8	R125r	0.15	30.2	0.01	90.0	0.1	98.5
R083o	0.03	54.0	0.31	6.0	11.7	100.0	R126r	0.14	36.2	0.01	74.2	0.1	97.5
R084o	0.01	14.5	0.11	8.5	12.1	100.0	R127r	0.16	20.4	0.01	68.5	0.1	99.6
R085o	0.03	123.3	0.42	12.3	12.3	99.9	R128r	0.06	31.2	0.01	70.0	0.1	98.3
R086o	0.03	64.4	0.41	3.0	12.9	100.0	R129r	0.04	4.0	0.00	137.9	0.1	99.9
R087o	0.03	100.1	0.42	9.7	13.5	100.0	R130r	0.89	23.4	0.10	141.4	0.1	98.9
R088o	0.08	64.0	1.04	11.8	13.6	100.0	R131r	0.09	37.5	0.01	96.8	0.1	96.6
R089o	0.02	59.6	0.26	42.5	13.8	96.3	R132r	0.23	21.2	0.03	61.3	0.1	99.4
R090o	0.03	34.2	0.42	30.4	14.0	98.7	R133r	0.05	20.9	0.01	45.0	0.1	99.5
R091o	0.01	98.3	0.15	30.2	14.3	98.7	R134r	0.05	19.0	0.01	87.8	0.1	99.4
R092o	0.00	86.7	0.03	16.5	14.9	99.8	R135r	0.08	17.8	0.01	85.1	0.1	99.5
R093o	0.03	37.8	0.45	15.1	15.2	99.9	R136r	0.19	18.0	0.03	89.1	0.1	99.4
R094o	0.02	74.8	0.32	23.7	16.0	99.5	R137r	0.53	20.5	0.08	89.0	0.2	99.1
R095o	0.01	85.5	0.23	34.8	17.4	98.1	R138r	0.47	10.6	0.08	63.2	0.2	99.9
R096o	0.03	48.1	0.57	12.1	18.1	100.0	R139r	0.24	36.1	0.04	73.0	0.2	96.2
R097o	0.01	51.3	0.25	16.7	18.2	99.9	R140r	0.11	27.8	0.02	64.0	0.2	98.2
R098o	0.01	106.3	0.13	32.6	18.2	98.4	R141r	0.04	15.6	0.01	131.8	0.2	98.3
R099o	0.01	54.3	0.10	42.5	19.0	96.5	R142r	0.17	7.0	0.03	42.6	0.2	100.0
R100o	0.00	141.4	0.01	40.6	19.3	96.9	R143r	0.14	6.9	0.03	24.9	0.2	100.0
R101o	0.03	30.5	0.51	6.4	20.3	100.0	R144r	0.20	24.4	0.04	52.0	0.2	98.7
R102o	0.01	81.6	0.21	16.3	20.4	99.9	R145r	0.02	27.9	0.00	72.2	0.2	97.8

R103o	0.00	97.9	0.10	20.4	23.7	99.7	R146r	0.06	10.6	0.01	76.7	0.2	99.6
R104o	0.00	131.9	0.04	45.5	25.7	95.9	R147r	0.11	23.7	0.02	67.5	0.2	98.3
R105o	0.01	89.8	0.15	48.0	27.7	95.3	R148r	0.34	9.3	0.08	44.4	0.2	99.9
R106o	0.00	141.4	0.02	41.1	31.4	97.0	R149r	0.37	20.2	0.09	40.0	0.2	99.1
R107o	0.02	126.8	0.55	18.3	34.8	99.8	R150r	0.21	12.3	0.05	15.6	0.2	99.9
R108o	0.00	123.3	0.08	40.0	40.5	97.4	R151r	0.07	15.0	0.02	41.3	0.3	99.6
R109o	0.01	135.5	0.23	13.2	40.6	99.9	R152r	0.12	8.0	0.03	59.4	0.3	99.7
R110o	0.00	84.3	0.03	45.4	44.0	96.2	R153r	0.12	21.9	0.03	43.9	0.3	98.7
R111o	0.01	79.6	0.47	13.1	47.3	100.0	R154r	1.34	11.5	0.35	49.2	0.3	99.6
R112o	0.01	119.5	0.45	27.5	84.0	99.3	R155r	0.34	12.4	0.09	48.5	0.3	99.6
R156r	0.28	23.5	0.07	41.4	0.3	98.4	R182r	0.10	19.1	0.04	23.6	0.4	98.6
R157r	0.18	27.8	0.05	30.3	0.3	97.7	R183r	0.21	2.2	0.08	58.1	0.4	98.3
R158r	0.27	20.6	0.07	33.0	0.3	99.0	R184r	0.07	17.9	0.03	20.8	0.4	98.8
R159r	0.13	14.9	0.06	41.8	0.5	95.0	R185r	0.99	11.7	0.41	23.5	0.4	99.5
R160r	0.23	3.4	0.06	71.1	0.3	99.4	R186r	0.22	21.3	0.09	10.0	0.4	98.2
R161r	0.75	11.6	0.21	66.0	0.3	99.1	R187r	0.05	29.2	0.02	5.0	0.4	95.1
R162r	0.22	27.2	0.06	18.8	0.3	97.9	R188r	0.30	13.7	0.13	5.0	0.4	99.6
R163r	0.24	24.7	0.07	35.4	0.3	98.1	R189r	0.30	11.0	0.13	4.2	0.4	99.8
R164r	0.52	20.8	0.15	13.6	0.3	99.1	R190r	0.15	10.0	0.07	42.9	0.4	97.9
R165r	0.13	24.4	0.04	26.8	0.3	98.3	R191r	0.41	17.4	0.18	18.6	0.4	98.5
R166r	0.07	5.0	0.02	47.9	0.3	99.7	R192r	0.29	1.6	0.13	25.5	0.4	99.8
R167r	0.28	11.8	0.09	28.2	0.3	99.7	R193r	0.14	13.0	0.06	8.6	0.4	99.6
R168r	0.29	21.7	0.09	17.7	0.3	98.7	R194r	0.29	4.1	0.13	28.9	0.5	99.5
R169r	0.21	3.4	0.07	27.8	0.3	99.9	R195r	0.17	6.4	0.08	24.9	0.5	99.6
R170r	0.09	31.0	0.03	30.1	0.3	95.7	R196r	0.44	7.6	0.20	21.5	0.5	99.7
R171r	0.52	15.7	0.17	73.5	0.3	96.9	R197r	0.12	4.3	0.05	31.6	0.5	99.3
R172r	0.89	4.8	0.30	35.0	0.3	99.8	R198r	1.67	18.1	0.76	29.8	0.5	97.2
R173r	0.06	27.1	0.02	16.6	0.3	97.3	R199r	0.45	8.7	0.21	24.2	0.5	99.4
R174r	0.22	16.4	0.07	11.9	0.3	99.5	R200r	0.12	11.8	0.06	8.2	0.5	99.6
R175r	0.22	26.4	0.08	50.6	0.4	95.5	R201r	0.19	4.8	0.09	33.4	0.5	98.9
R176r	0.25	9.8	0.09	40.2	0.4	99.4	R202r	0.35	5.8	0.17	27.1	0.5	99.3
R177r	0.59	29.1	0.21	30.4	0.4	95.7	R203r	0.21	15.7	0.11	24.6	0.5	97.7
R178r	0.28	15.8	0.10	18.5	0.4	99.4	R204r	0.54	12.4	0.27	19.4	0.5	98.9
R179r	0.47	23.8	0.17	24.7	0.4	97.7	R205r	0.39	10.2	0.19	6.8	0.5	99.7
R180r	0.56	12.3	0.21	32.9	0.4	99.4	R206r	0.87	10.5	0.44	14.5	0.5	99.5
R181r	1.02	1.7	0.37	36.6	0.4	99.7	R207r	0.87	10.5	0.44	14.5	0.5	99.5

Table 4

Regulated protein spots after exposure of 3 days old zebrafish eleutheroembryos for a duration of 48 h to the EC_{10} concentration of DNOC. 76 spots changed in expression. 56 of these protein spots were up-regulated and 20 down-regulated.

Spot Nr.	Mean C	Rsd [%]	Mean EC_{10}	Rsd [%]	Ratio	t-Test	Spot
34	0.01	119.7	0.06	26.6	10.81	98.88	D34o
35	0.01	141.4	0.07	33.1	11.98	97.90	D35o
36	0.00	141.4	0.02	32.9	12.40	97.99	D36o
37	0.00	82.8	0.09	11.6	26.09	99.97	D37o
38	0.00	139.6	0.09	15.7	41.79	99.90	D38o
39	0.15	23.6	0.32	6.0	2.10	99.57	D39o
40	0.04	33.6	0.13	18.5	3.11	98.90	D40o
41	0.06	20.0	0.18	10.0	3.13	99.87	D41o
42	0.11	41.0	0.40	4.0	3.59	99.89	D42o
43	0.08	50.2	0.32	17.0	3.81	99.17	D43o
44	0.08	98.2	0.34	14.9	4.17	98.18	D44o
45	0.05	60.2	0.23	24.1	4.21	98.17	D45o
46	0.02	57.5	0.08	30.2	4.61	97.26	D46o
47	0.03	59.2	0.15	35.0	4.81	96.08	D47o
48	0.06	76.4	0.34	21.8	6.09	99.07	D48o
49	0.04	64.8	0.27	26.0	6.91	98.81	D49o
50	0.01	6.5	0.11	19.1	8.04	99.71	D50o
51	0.01	67.7	0.09	27.6	10.30	98.92	D51o
52	0.01	62.8	0.12	25.1	10.68	99.23	D52o
53	0.02	85.9	0.06	23.5	3.41	95.60	D53o
54	0.01	86.0	0.03	1.2	3.43	96.76	D54o
55	0.04	76.7	0.16	26.3	3.67	96.26	D55o
56	0.02	99.0	0.10	18.8	3.91	97.11	D56o
57	0.05	67.3	0.20	7.5	4.03	99.56	D57o
58	0.13	34.4	0.27	8.1	2.04	98.15	D58o
59	0.00	95.0	0.02	43.8	32.69	96.45	D59o
60	0.03	32.8	0.06	13.9	2.07	97.41	D60o
61	0.03	31.3	0.09	5.7	3.24	99.90	D61o
62	0.20	32.8	0.43	6.9	2.18	99.01	D62o
63	0.22	11.7	0.48	19.4	2.19	98.14	D63o
64	0.17	41.8	0.37	15.5	2.25	96.88	D64o
65	0.03	42.0	0.07	11.1	2.26	97.80	D65o
66	0.16	44.7	0.36	12.3	2.30	97.42	D66o
67	0.39	36.7	0.92	23.9	2.36	95.38	D67o
68	0.07	47.8	0.17	3.6	2.36	98.34	D68o
69	0.08	51.6	0.25	31.5	3.29	95.10	D69o
70	0.01	43.9	0.04	14.5	2.75	98.59	D70o
71	0.11	74.5	0.29	12.0	2.75	96.13	D71o

72	0.04	52.1	0.12	20.0	2.85	97.23	D72o
73	0.02	73.3	0.07	18.0	2.86	95.71	D73o
74	0.10	53.8	0.30	5.7	2.90	99.12	D74o
75	0.04	73.5	0.12	19.9	3.06	96.20	D75o
76	0.08	90.2	0.25	15.7	3.07	95.41	D76o
77	0.02	53.5	0.07	23.9	3.07	96.79	D77o
78	0.02	82.2	0.06	20.7	3.12	95.46	D78o
79	0.04	19.1	0.13	12.0	3.16	99.80	D79o
80	0.10	60.0	0.44	20.9	4.32	98.77	D80o
81	0.07	49.9	0.34	13.8	4.61	99.68	D81o
82	0.04	67.9	0.17	33.6	4.62	96.09	D82o
83	0.04	32.9	0.19	25.4	5.26	98.82	D83o
84	0.02	123.8	0.10	24.5	5.30	97.24	D84o
85	0.07	62.5	0.35	23.5	5.31	98.80	D85o
86	0.01	98.2	0.08	29.2	6.02	97.57	D86o
87	0.01	85.1	0.04	28.1	7.57	98.46	D87o
88	0.00	99.4	0.04	17.1	10.81	99.73	D88o
89	0.00	117.9	0.04	16.7	32.41	99.87	D89o
95	0.02	30.5	0.00	86.4	0.00	99.01	D95r
96	0.07	22.4	0.02	75.2	0.27	97.28	D96r
97	0.11	14.1	0.04	76.1	0.32	97.28	D97r
98	0.10	6.0	0.03	59.3	0.34	98.90	D98r
99	0.06	16.8	0.02	71.3	0.37	95.52	D99r
100	0.07	21.9	0.03	48.5	0.39	95.92	D100r
101	0.23	12.2	0.11	15.9	0.48	99.30	D101r
102	0.03	36.8	0.00	65.4	0.05	97.78	D102r
103	0.04	21.6	0.01	71.1	0.17	99.06	D103r
104	0.07	39.8	0.01	3.7	0.18	95.60	D104r
105	0.26	27.3	0.07	57.9	0.25	97.33	D105r
106	0.23	27.5	0.07	59.9	0.31	95.90	D106r
107	0.18	29.4	0.06	32.7	0.35	95.69	D107r
108	0.07	18.7	0.03	41.9	0.39	97.42	D108r
109	0.20	19.8	0.09	42.1	0.42	96.30	D109r
110	0.20	9.2	0.09	27.5	0.45	99.25	D110r
111	0.12	19.4	0.06	30.5	0.47	96.47	D111r
112	0.51	17.4	0.25	30.6	0.48	96.82	D112r
113	0.32	9.3	0.15	19.8	0.49	99.43	D113r
114	0.09	15.9	0.04	36.1	0.49	96.13	D114r

Table 5

Regulated spots after exposure of 3 days old zebrafish eleutheroembryos for a duration of 48 h to the EC_{10} concentration of diclofenac. 47 spots changed in expression. 42 of these protein spots were up-regulated and 5 down-regulated.

Spot Nr.	Mean C	Rsd [%]	Mean EC_{10}	Rsd [%]	ratio	t-Test	Spot
11	0.13	39.8	0.32	17.6	2.4	97.4	Dic11o
12	0.06	21.7	0.13	1.8	2.3	99.9	Dic12o
13	0.04	16.4	0.14	35.8	3.7	95.4	Dic13o
14	0.04	60.3	0.13	12.9	3.0	98.2	Dic14o
15	0.04	48.9	0.13	21.8	3.7	98.5	Dic15o
16	0.01	70.5	0.07	9.1	5.9	99.9	Dic16o
17	0.02	61.6	0.10	5.1	4.7	99.9	Dic17o
18	0.00	83.1	0.06	21.6	18.3	99.6	Dic18o
19	0.00	77.7	0.09	9.9	21.5	100.0	Dic19o
20	0.00	33.3	0.13	21.8	33.8	99.7	Dic20o
21	0.02	68.4	0.06	10.3	4.0	99.4	Dic21o
22	0.02	19.3	0.05	23.8	2.1	95.3	Dic22o
23	0.08	28.0	0.18	13.9	2.4	98.9	Dic23o
24	0.05	13.0	0.11	12.0	2.0	99.4	Dic24o
25	0.03	51.2	0.07	13.6	2.2	95.3	Dic25o
26	0.06	38.9	0.12	10.6	2.1	97.7	Dic26o
27	0.08	26.2	0.18	17.0	2.3	98.2	Dic27o
28	0.04	20.9	0.10	9.6	2.3	99.7	Dic28o
29	0.05	19.8	0.15	9.8	2.8	99.8	Dic29o
30	0.03	31.7	0.12	26.2	3.4	97.6	Dic30o
31	0.08	35.3	0.23	25.7	2.7	96.4	Dic31o
32	0.03	52.6	0.06	12.1	2.5	97.5	Dic32o
33	0.03	43.9	0.08	25.3	2.7	95.8	Dic33o
34	0.02	68.2	0.06	17.9	4.0	98.7	Dic34o
35	0.02	73.7	0.08	27.4	3.6	96.0	Dic35o
36	0.03	16.5	0.09	28.7	3.2	97.1	Dic36o
37	0.00	136.8	0.03	28.8	36.8	99.1	Dic37o
38	0.00	9.7	0.01	21.2	3.2	98.9	Dic38o
39	0.02	66.4	0.12	21.2	5.1	99.0	Dic39o
40	0.01	90.5	0.05	24.4	5.3	98.2	Dic40o
41	0.01	68.1	0.04	28.0	4.4	97.3	Dic41o
42	0.00	122.5	0.17	38.1	44.9	97.8	Dic42o
43	0.00	75.5	0.01	39.4	14.2	97.0	Dic43o
44	0.01	81.9	0.03	15.8	5.8	99.5	Dic44o
45	0.00	55.9	0.01	24.8	9.9	99.2	Dic45o
46	0.00	141.4	0.03	36.9	20.6	97.7	Dic46o
47	0.00	94.9	0.04	32.4	26.5	98.6	Dic47o
48	0.00	141.4	0.03	31.4	39.2	98.8	Dic48o

49	0.00	0.00	0.01	11.7	∞	100.0	Dic49o
50	0.00	0.00	0.02	35.4	∞	98.4	Dic50o
51	0.17	35.1	0.45	10.2	2.7	99.4	Dic51o
52	0.02	10.7	0.01	79.0	0.3	96.6	Dic52o
53	0.09	11.9	0.08	53.0	0.9	30.1	Dic53r
54	0.12	3.4	0.06	27.9	0.5	99.2	Dic54r
55	0.03	26.4	0.00	0.00	0.0	99.4	Dic55r
56	0.90	17.6	0.39	40.4	0.4	96.8	Dic56r
57	0.12	18.8	0.05	51.4	0.4	96.3	Dic57r
58	0.03	21.2	0.01	52.2	0.2	98.8	Dic58r
59	0.04	14.7	0.01	49.4	0.3	98.8	Dic59r
60	0.06	10.1	0.03	40.3	0.5	97.4	Dic60r
61	0.19	25.4	0.09	13.8	0.5	95.3	Dic61r
62	0.19	23.1	0.09	3.9	0.5	97.1	Dic62r
63	0.12	19.3	0.06	38.2	0.5	95.7	Dic63r
64	0.17	8.5	0.08	15.4	0.5	99.7	Dic64r
65	0.39	5.5	0.19	18.1	0.5	99.8	Dic65r
66	0.09	18.0	0.04	18.1	0.4	98.4	Dic66r
