# Supplementary material for: Proteomic Signatures of the Zebrafish (Danio rerio) Embryo: Sensitivity and Specificity in Toxicity Assessment of Chemicals
Source: Int J Proteomics. 2010 Oct 14;2010:630134. doi: 10.1155/2010/630134 (PMC3200224; doi:10.1155/2010/630134)
Supplement: Supplementary file 2 [file 630134.f2.pdf]

Supplementary Material: Figure SM1

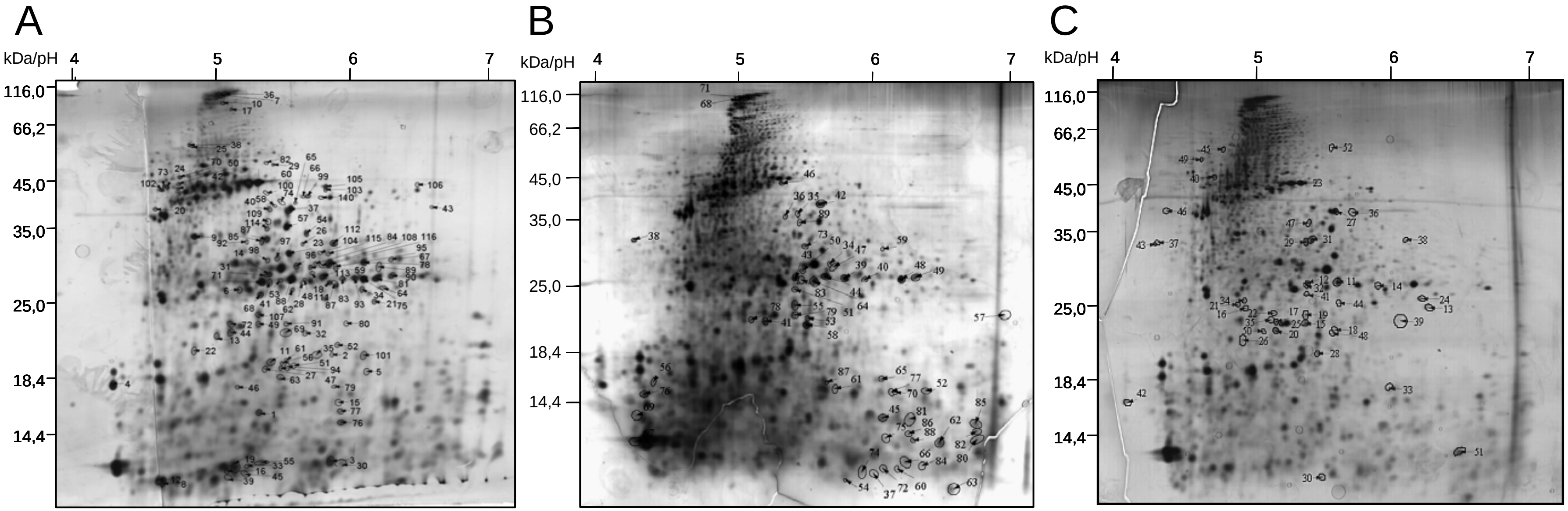

Figure SM1

Comparison of the proteome pattern of eleutheroembryos of the zebrafish exposed to rotenone (A) DNOC (B) and diclofenac (C) with the respective EC_{10} concentration (rotenone = 0.05 µM; DNOC = 2.80 µM; Diclofenac = 12,6 µM). Only in expression upregulated proteins are depicted. The x-axis shows the pH-range and the y-axis the molecular weight (MW) in kDa. Exposed to rotenone, 116 proteins, to DNOC, 56 proteins and to Diclofenac, 41 proteins are increased in their intensity. Modified proteins are encircled and labelled with numbers. The labelled spots can be found in the supplementary material section.

Supplementary Material: Figure SM2

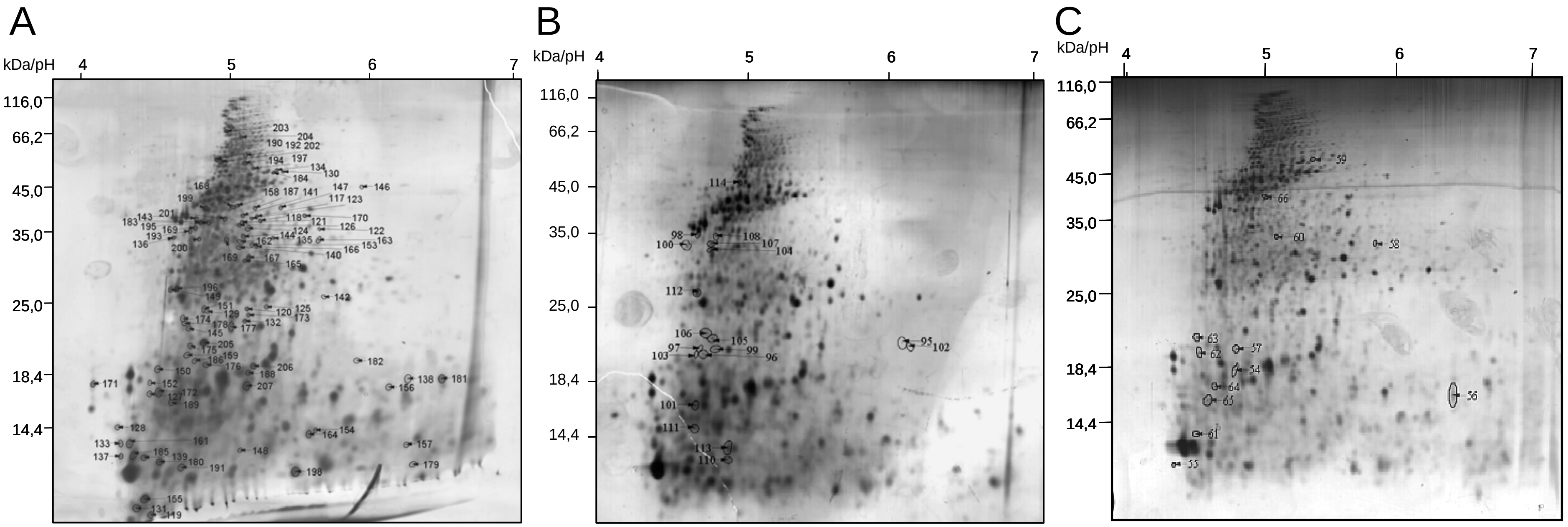

Figure SM2

Comparison of the proteome pattern of eleutheroembryos of the zebrafish exposed to rotenone (A), DNOC (B) and diclofenac (C) with the respective EC_{10} concentration (rotenone = 0.05 µM; DNOC = 2.80 µM; diclofenac = 12,6 µM). Only repressed proteins are depicted. The x-axis shows the pH-range and the y-axis the molecular weight (MW) in kDa. Exposed to rotenone, 91 proteins, to DNOC, 20 proteins and to diclofenac, 14 proteins are decreased in their intensity. Modified proteins are encircled and labelled with numbers. The labelled spots can be found in supplementary material section.
